# Improved method for qualitative screening of lipolytic bacterial strains

**DOI:** 10.1016/j.mex.2018.01.004

**Published:** 2018-02-01

**Authors:** Jair Carrazco-Palafox, Blanca E. Rivera-Chavira, Norma Ramírez-Baca, Luisa I. Manzanares-Papayanopoulos, Guadalupe V. Nevárez-Moorillón

**Affiliations:** a Facultad de Medicina y Ciencias Biomédicas, Universidad Autónoma de Chihuahua, Circuito No. 1, Campus Universitario II Chihuahua, Chihuahua 31125, Mexico; b Facultad de Ciencias Químicas, Universidad Autónoma de Chihuahua, Circuito Universitario s/n, Campus Universitario II, Chihuahua, Chihuahua 31125, Mexico; c Secretaría de Comunicaciones y Obras Públicas, Gobierno del Estado de Chihuahua, Beethoven 4000, Fraccionamiento la Herradura, Chihuahua, Chihuahua 31206, Mexico; d Capítulo Mexicano del Consejo Empresarial Mundial para el Desarrollo Sostenible, Comisión de Estudios del Sector Privado para el Desarrollo Sustentable, Lancaster 15, Col. Juárez, Mexico City, D.F. 06600, Mexico

**Keywords:** Improved method for qualitative screening of lipolytic bacterial strains, Lipase activity, Calcium ion, Magnesium ion, Bioprospecting

## Abstract

Esterases and lipases are lipolytic enzymes that catalyse the hydrolysis of triacylglycerols, Determination of lipolysis on agar plates is a simple approach to determine lipase or esterase action, but visual evaluation of lipolysis is frequently difficult in practice. Therefore, the aim of this work was to improve the efficiency of lipolysis visualization in tributyrin agar (mTBA) by adding calcium and/or magnesium ions in the screening of lipolytic microbial strains. Lipolytic activity was evaluated in mTBA using the well diffusion technique, where a clear zone around the inoculated wells indicated lipid hydrolysis. Results suggest that the addition of 2.5 mM calcium and 5.0 mM magnesium was the best combination of ion addition to TBA. Lipolytic activity increased the clearing zone up to 38% more than without the addition of ions and the clear zone was clearly observed. The mTBA plate was used with culture collection microbial strains, as well as with a collection of soil microorganisms, to identify lipase producers. The addition of calcium and magnesium ions can provide an easier screening procedure for selection of lipolytic bacterial strains.

• A modified tributyrin agar for screening of lipolytic bacteria was prepared by adding calcium and magnesium ions.
• The modified TBA agar was tested with control bacterial strains, and, based on the results, 2.5 mM Ca and 5.0 mM Mg ions were added in the mTBA.
• mTBA was validated with environmental bacterial strains for screening of lipolytic activity.

## Graphical abstract

**Figure:**
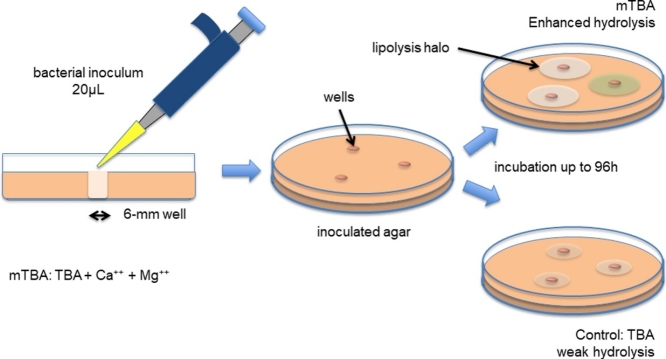

## Method details

### Background

Esterases (E.C. 3.1.1.1) and lipases (E.C. 3.1.1.3) are lipolytic enzymes that catalyse the hydrolysis of triacylglycerols [[1], [2], [3]]. Lipase hydrolytic activity has been measured by titration, spectroscopy, chromatography, radioactivity, tensiometry, turbidimetry, conductimetry, immunochemistry, and microscopy. These techniques can be employed in the detection of fatty acids or glycerol release from the triacylglycerols or fatty acid esters. Additionally, lipolysis measure is an important approach to determine the lipase action as well as the change of product properties at the interface [[4], [5], [6], [7]].

As part of the biochemical characterization of lipase and esterase enzymes, the effect of environmental factors, such as pH, temperature, and presence of metallic ions have been studied [[8], [9], [10], [11]], the result of those studies has been used for establishing culture conditions for enzyme production [[12], [13]]. Determination of lipolysis in bacterial isolates is usually evaluated on agar plates [7], such as tributyrin agar (TBA) or spirit blue agar. TBA is the most commonly used media for the screening of lipolytic strains by the development of a clear zone around the colony, as indicative of lipase or esterase activity [[7], [9], [14]]. However, visual evaluation of lipolysis is frequently difficult in practice. Therefore, the aim of this work was to improve the use of tributyrin agar used in screening of lipolytic microbial strains by adding divalent ions to enhance medium stability [[14], [15]].

## Experimental procedures

Bacterial strains were isolated from environments located in northern Mexico. These isolates were exposed to soils contaminated with burnt oils. A total of 98 isolates were collected and then analysed for the lipolytic activity. Strains were maintained on tripticasein soy agar (Bioxon, Becton, Dickinson and Company, Franklin Lakes, NJ) at room temperature. They were sub-cultured in tripticasein soy broth (BD) at 37 °C for 20–24 h before use. *Pseudomonas aeruginosa* ATCC 27853, *Bacillus subtilis* ATCC 6633, *Staphylococcus aureus* ATCC 6538, and *Proteus mirabilis* ATCC 12453 were used as control bacteria strains. Control bacterial strains were maintained under the same conditions.

Culture agar was TBA, containing 15 g bacteriological agar (BD), 3 g yeast extract (BBL, Mexico), 5 g peptone (BBL, Mexico), tributyrin 1% v/v (Sigma-Aldrich, St. Louis, MO), modified by adding Ca^++^ (CaCl_2_) and/or Mg^++^ (MgSO_4_). A full factorial design, with concentrations of calcium and magnesium ions (0.0 mM, 2.5 mM, 5.0 mM, and 10.0 mM in distilled water) was used to test the efficiency of metallic ions on mTBA performance.

Lipolytic activity was carried out on mTBA in 0.6 cm diameter wells; the wells were cut-off using sterilised glass Pasteur pipettes. The strains were subsequently inoculated in the wells by placing a 20 μL aliquot of a 24 h bacterial culture (1 − 2 × 10^7^ cells approximately). All plates were incubated at 28 °C and lipolytic hydrolysis was measured as a clear zone around the inoculated wells. Lipolytic activity data was measured in 22 replicates for each treatment and control; the diameter of the clear zone or halo was measured as a lipolysis indicator. The halos were measured at 12, 24, and 48 h. Small variations of TBA consistency was observed during preparation of plates that can be attributed to the concentration of the metallic ions present. Overall, the firmer the medium consistency, the better was the observation of lipolytic activity. All data were analysed using the SAS software by ANOVA for factorial design [16].

## Validation

Previous studies on screening of lipid degrading bacteria [[7], [9], [14]] used the development of a clear zone around the colony as indicative of lipase or esterase activity. TBA was the most commonly used agar. Culture medium was modified by adding ions of Fe^++^, Mg^++^, and Ca^++^ to enhance lipase production in *Ps. aeruginosa* [17].

In our study, all control strains showed lipolytic activity with or without the addition of calcium and/or magnesium ions. However, lipolytic activity was significantly different among the bacterial strains tested (Table 1). Those differences could be attributed to bacterial strains and the nature of their lipases.

**Table 1:** Lipolytic activity of control strains on the modified TBA, measured as clear zones (mm).

*Pseudomonas aeruginosa* ATCC27853	1.73^a^
*Staphylococcus aureus* ATCC 6538	1.60^b^
*Bacillus subtilis* ATCC 6633	1.58^b^
*Proteus mirabilis* ATCC12453	1.42^c^

Different letters represent different groups, as determined by Tukey test (*P* < 0.05).

*Ps. aeruginosa* ATCC 27853 showed the highest lipase activity of all control strains tested (Table 1). The diameter of the clear zone affected by concentrations of both calcium and magnesium are presented in Table 2. *Ps. aeruginosa* lipolytic activity was increased by a single addition of 2.5 mM calcium (F = 18.37, *P <* 0.001), or 5.0 mM magnesium (F = 5.84, *P <* 0.001). 2.5 mM calcium and 5.0 mM magnesium showed the highest interaction. The clear zone of 2.02 cm was largest with *Ps. aeruginosa*. The smallest intensity of agar clearing by lipolysis was with the unmodified medium (Table 2).

**Table 2:** Lipolytic activity in terms of zone of hydrolysis (in mm) in tributyrin agar by diffusion test in wells.

		Calcium (mM)			
		0.0	2.5	5.0	10.0
	*Pseudomonas aeruginosa* ATCC 27853				
Magnesium (mM)	0.0	1.46 ± 0.33 ^a,2^	1.83 ± 0.26 ^a,1^	1.65 ± 0.51 ^b,12^	1.47 ± 0.39 ^a,2^
	2.5	1.46 ± 0.33 ^a,3^	1.96 ± 0.34 ^a,1^	1.83 ± 0.27 ^ab,12^	1.71 ± 0.33 ^b,2^
	5.0	1.65 ± 0.21 ^a,2^	2.02 ± 0.35 ^a,1^	1.75 ± 0.40 ^ab,2^	1.83 ± 0.31 ^b,12^
	10.0	1.55 ± 0.47 ^a,2^	1.81 ± 0.32 ^a,1^	1.90 ± 0.31 ^a,1^	1.75 ± 0.30 ^b,1^
	*Staphylococcus aureus* ATCC 6538				
	0.0	1.65 ± 0.37 ^a,1^	1.61 ± 0.30 ^a,1^	1.57 ± 0.38 ^a,1^	1.65 ± 0.29 ^a,1^
	2.5	1.76 ± 0.19 ^a,1^	1.30 ± 0.25 ^b,2^	1.64 ± 0.16 ^a,1^	1.69 ± 0.19 ^a,1^
	5.0	1.61 ± 0.35 ^a,1^	1.67 ± 0.21 ^a,1^	1.62 ± 0.23 ^a,1^	1.63 ± 0.12 ^ab,1^
	10.0	1.45 ± 0.26 ^b,3^	1.64 ± 0.18 ^a,12^	1.68 ± 0.18 ^a,1^	1.49 ± 0.27 ^b,23^
	*Bacillus subtilis* ATCC 6633				
	0.0	1.50 ± 0.18 ^a,1^	1.46 ± 0.27 ^c,1^	1.54 ± 0.34 ^b,1^	1.49 ± 0.47 ^b,1^
	2.5	1.44 ± 0.26 ^ab,2^	1.70 ± 0.25 ^b,1^	1.70 ± 0.21 ^a,1^	1.65 ± 0.17 ^a,1^
	5.0	1.45 ± 0.24 ^ab,2^	1.60 ± 0.22 ^bc,12^	1.64 ± 0.14 ^ab,1^	1.67 ± 0.16 ^a,1^
	10.0	1.32 ± 0.42 ^b,3^	1.87 ± 0.13 ^a,1^	1.67 ± 0.08 ^ab,2^	1.58 ± 0.18 ^ab,2^
	*Proteus mirabilis* ATCC 12453				
	0.0	1.26 ± 0.36 ^b,2^	1.49 ± 0.39 ^ab,1^	1.54 ± 0.39 ^a,1^	1.41 ± 0.45 ^a,12^
	2.5	1.30 ± 0.20 ^b,2^	1.43 ± 0.11 ^b,12^	1.52 ± 0.19 ^a,1^	1.39 ± 0.13 ^a,12^
	5.0	1.57 ± 0.42 ^a,1^	1.64 ± 0.15 ^a,1^	1.50 ± 0.28 ^a,1^	1.15 ± 0.36 ^b,2^
	10.0	1.24 ± 0.53 ^b,2^	1.44 ± 0.19 ^b,1^	1.52 ± 0.18 ^a,1^	1.38 ± 0.31 ^a,12^

The results are the mean ± SD of 22 replicates. Different letters for each microorganism represent different groups, as determined by the Tukey test (*p <* 0.05). Different numbers in each row for each microorganism represent different groups, as determined by the Tukey test (*p <* 0.05).

*Staphylococcus aureus* is known to have lipolytic activity [[3], [8], [18], [19]]. At the same time, activity of this strain resembles *Bacillus subtilis*, but different than the activities of *Ps. aeruginosa* and *P. mirabilis*, according to statistical analysis (Table 1). Lipolytic activity of *S. aureus* increased considerably by adding 2.5 mM of magnesium. However, the same magnesium concentration was drastically affected by 2.5 mM calcium (Table 2). This result could be the consequence of differences in microbial metabolism and its tolerance to positive or negative ions [15].

Although, *B. subtilis* showed the same statistical analysis as *S. aureus*, the increase in the concentration of magnesium alone had a slightly negative effect on lipolytic activity; the presence of 2.5 mM calcium increased the clear zone up to 1.87 cm when the magnesium concentration was 10.0 mM.

*P. mirabilis* ATCC 12453 showed the lowest lipolytic activity of all control strains (Table 1). Nevertheless, the lipolytic activity of *P. mirabilis* increased considerably by the interaction of calcium 2.5 mM and magnesium 5.0 mM (F = 3.371, *P <* 0.001). The most stable results occurred with 5.0 mM calcium ion at any concentration of magnesium.

The low lipolytic activity of lipolytic strains was enhanced by the media modified with 2.5 mM calcium and 5.0 mM magnesium. Based on these results with standard control strains, these concentrations were selected for the detection of lipase activity in a bacterial strain collection of environmental isolates from soils contaminated with hydrocarbon. A total of 98 isolates were tested and after an initial screening, 20 strains were selected for their lipolytic activity. The lipolysis assay was carried out as described for control strains, with lipolysis clear zone measurements at 24, 48, 72, and 96 h of incubation.

**Fig. 1:**
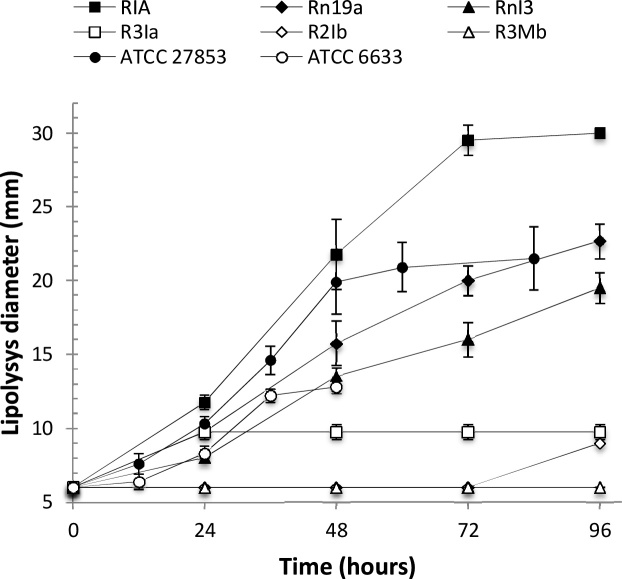
Lipolysis kinetics of strains RIA, Rn19a, RnI3, R3Ia, R2Ib, and R2Mb, compared with the control strains, *Pseudomonas aeruginosa* ATCC 27853 and *Bacillus subtilis* ATCC 6633. Culture media was mTBA with 2.5 mM calcium and 5.0 mM magnesium added.

Of the 20 strains that showed positive lipolytic capacity on the TBA medium, only six representative samples were selected for retesting on the mTBA medium. The strains were the ones marked as RIA, Rn19a, RnI3, R3Ia, R2Ib, and R2Mb, all of which were identified by biochemical tests as *Pseudomonas*. Also, they were contrasted with control strains, *Ps. aeruginosa* ATCC 27853 (well known as a highly lipolytic strain) and *B. subtilis* ATCC 6633 (previously, described as the poorest lipolytic strain, but enhanced by adding divalent ions). Fig. 1 shows the results obtained, using the mTBA.

The lipolysis of the RIA, Rn19a, and RnI3 strains was significantly higher than the strains R3Ia, R2Ib, and R2Mb. The group RIA, Rn19a, and RnI3 resembled the control ATCC 278553. Of this group, RIA stands out for the highest measure of lipolysis hydrolysis. The strains clustered in the group including R3Ia, R2Ib, and R3Ia resembled the control *B. subtilis* ATCC 6633, that showed a discrete but clear lipolysis. The strains R3Ia, R2Ib, and R2Mb had values lower than *B. subtilis*. R2Ib had the poorest and delayed lipolysis, which was visible after 96 h of incubation. R3Mb had no lipolytic activity under the tested conditions. Strains R2Ib and R3Mb had lipolytic activity under other conditions (i.e., ion concentrations and time). Lipolytic activities, as well as the differences in magnitude, were clearly observed after 48 h. For the purposes of the culture medium, we propose that most of the bacterial strains increased their lipolytic activity.

We observed, that at 48 h of incubation and later on, strain Rn19a had a smaller halo of dark green/brown pigmentation around the clear zone. After 48 h of incubation, strain RIA had a yellowish pigmentation over the halo. These pigmentations did not represent a problem for measuring lipolytic activity, but we consider that this observation can be important for further studies on the differential expression of pigments, since it might have a positive effect on lipolysis, including association with bio-surfactants [[20], [21], [22]].

## Discussion

Detection of lipase activity on environmental isolates has always been a problem because of the lack of appropriate screening methods [[19], [23]]. The calcium ion has been reported as the main ion that increases lipase enzymatic activity, while magnesium has also been reported as an enhancer of lipase activity for *in vitro* tests of some *Pseudomonas* species [[9], [20], [24]]. These reports are in opposition to Bisht et al. [17], since they assure that lipases do not require cofactors.

According to some reports [[9], [25]], tributyrin agar is a medium for strain selection, and results should be determined between 72 h and up to 7 days of incubation [26]. Other authors used other media to detect lipolytic activity on plates, including the use of different substrates, which is not always useful for all microbial species [[13], [25], [27]].

*Pseudomonas* strains have been used extensively for lipase production and Ca^++^ has activated its hydrolysis; whereas Zn^++^, Fe^++^, and Al^+3^ inhibited hydrolysis of lipids [18]. Taking in consideration the results obtained in our work, calcium ion helps as an intensifier of lipolytic activity, while magnesium provides stability, when added to TBA. These interactions are supported by others [[28], [29]] who consider most halotolerant bacteria as having important enzymatic activities, such as with amylases and lipases. *P. mirabilis* was the only strain we studied that had not been reported as lipolytic in previous studies [[8], [9], [27], [30]].

Most of the *Pseudomonas* strains evaluated by other authors had good growth at 30 °C and formation of a clear zone of lipolytic activity after 72 h of incubation [[25], [27]]. Adding divalent ions to TBA can reduce the incubation time for screening of lipolytic bacteria. Incubation time on mTBA was reduced from seven days to 48–72 h. An interesting observation was that added divalent ions enhanced production of bacterial pigments.

## Conclusion

Adding 2.5 mM calcium and 5.0 mM magnesium to the TBA medium intensified lipolytic activity and increased the clear zone up to 38% more than the control TBA medium. Therefore, mTBA is a better culture medium for screening and identification of lipolytic bacterial strains. Future studies should focus on the diversity of the microbial communities where lipase-producing bacteria are present.
